# Increased expression of heparanase in osteogenic differentiation of rat marrow stromal cells

**DOI:** 10.3892/etm.2013.1070

**Published:** 2013-04-19

**Authors:** QINGLIN HAN, FAN LIU, YOULANG ZHOU

**Affiliations:** 1 Departments of Orthopedics, Affiliated Hospital of Nantong University, Nantong, Jiangsu 226001, P.R. China; 2 Hand Surgery, Affiliated Hospital of Nantong University, Nantong, Jiangsu 226001, P.R. China

**Keywords:** heparanase, marrow stromal cells, osteogenic differentiation

## Abstract

Heparanase (HPSE) is a type of endoglycosidase that decomposes the heparan sulfate (HS) lateral chains of heparan sulfate proteoglycans (HSPGs), releases related growth factors and participates in angiogenesis and bone formation. HPSE is expressed in osteoblasts and is involved in fracture healing. However, the role of HPSE in osteogenic differentiation requires in-depth investigation. To investigate the expression of HPSE in the osteogenic differentiation of rat marrow stromal cells (MSCs), the protein and mRNA expression levels of HPSE on days 0, 1, 3, 7, 10, 14 and 21 of osteogenic differentiation of MSCs in 2- and 10-month-old rats were detected using western blotting and reverse transcription-polymerase chain reaction (RT-PCR), respectively. From the third day of osteogenic differentiation onwards, all HPSE protein and mRNA expression levels in 2-month-old rats were significantly increased compared with basal levels (days 0 and 1; P<0.05). The protein and mRNA expression levels reached a peak on days 10 and 14, respectively, followed by a gradual decline. The same pattern was observed in 10-month-old rats; however, when compared with with basal levels, the differences were not statistically significant (P>0.05). The protein and mRNA levels of HPSE in the 2-month-old rats were significantly higher compared with the respective levels in the 10-month-old rats (P<0.05). HPSE is involved in the osteogenic differentiation of rat MSCs. The protein and mRNA expression levels of HPSE in aged rats are weaker compared with those in young rats, which may be related to the declined osteogenic differentiation ability.

## Introduction

Marrow stromal cells (MSCs) are the main source of osteogenic cells in fracture healing and bone tissue reconstruction (1). Under the action of related growth factors, MSCs gradually differentiate into osteogenic cells and synthesize extracellular matrix, including collagen, and ultimately transform into mature bone tissue (2). Number and function abnormalities of cytokines and growth factors directly cause weak differentiation of MSCs to osteoblasts, leading to osteoporosis and fracture healing disorders (3). A large amount of growth factors, including bone morphogenetic protein (BMP), fibroblast growth factor (FGF), vascular endothelial growth factor (VEGF) and transforming growth factor-β (TGF-β) in the extracellular matrix and on cell membrane surfaces are ingested and controlled by the heparan sulfate (HS) lateral chains of heparan sulfate proteoglycans (HSPGs) (4). HSPGs are widely distributed in the cytolemma and extracellular matrix, and the HS lateral chain has good affinity with growth factors (5). In physiological and pathological conditions, HSPGs upregulate the release and activity of the above growth factors and participate in extracellular matrix reconstruction, information transfer and signal transduction (6). It has been identified that HSPGs are involved in the regulation of endochondral ossification, bone tissue reconstruction and fracture healing (7).

Heparanase (HPSE) is a unique endoglycosidase that decomposes HS lateral chains of HSPGs and is closely related to the function of HSPGs. It not only plays a significant role in tumor metastasis and angiogenesis (8–10), but also participates in fracture healing (11) and bone tissue formation (12). HPSE is expressed in normal human osteoblasts (12). Whether HPSE is expressed and plays a role in the osteogenic differentiation of MSCs (precursor cells of osteoblasts) and whether the osteogenic differentiation of MSCs is regulated by intervention of HPSE expression, requires further investigation. In the current study, the protein and mRNA expression levels of HPSE in the osteogenic differentiation of rat MSCs were detected by western blot analysis and reverse transcription-polymerase chain reaction (RT-PCR), respectively. The aim of this study was to provide a foundation for further study of HPSE.

## Materials and methods

### Reagents and animals

The HPSE primary antibody was purchased from Santa Cruz Biotechnology, Inc. (Santa Cruz, CA, USA). TRIzol was provided by Invitrogen Life Technologies (Carlsbad, CA, USA). Alkaline phosphatase (ALP) and an alizarin red staining kit were purchased from Wuhan Boster Biological Technology, Ltd. (Wuhan, China). Three male 2-month-old Sprague-Dawley (SD) rats and three male 10-month-old SD rats were provided by the Experimental Animal Center of Nantong University.

### MSC separation and induced osteogenic differentiation

Single-cell suspensions of rat femur marrow were prepared under aseptic conditions. The conventional adherent culture method was used to separate and culture the MSCs. The cell morphology was observed under an inverted microscope. The second generation of MSCs was cultured in primary medium for 72 h, followed by osteogenic induction (10^−8^ mol/l dexamethasone, 10 mmol/l β-glycerophosphate sodium and 50 *μ*g/ml vitamin C). After 1 week, the cell morphology was observed under an inverted microscope. According to the manufacturer’s instructions, the ALP activity in MSCs was determined and alizarin red staining was conducted.

### Determination of HPSE protein expression by western blot analysis

Expression of the HPSE protein was determined using western blotting on days 0, 1, 3, 7, 10, 14 and 21 of osteogenic differentiation. Cell protein was extracted by the conventional method, followed by 10% sodium dodecyl sulfate-polyacrylamide gel electrophoresis (SDS-PAGE; 15 *μ*l sample in each well; stacking gel, 80 V, 20 min; separating gel, 120 V, 60 min). The wet electrical transfer method was used to transfer the protein on separating gel to a polyvinylidene fluoride (PVDF) membrane (constant voltage, 100 V for 90 min). Following Ponceau staining, the clear red bands in the PVDF membrane indicated the successful transfer. The PVDF membrane was immersed in 5% milk powder solution at room temperature for 2 h of blocking. The primary antibody with different dilution ratios was added, followed by incubation at 4°C overnight. After washing with Tris-buffered saline with Tween-20 (TBST) containing 0.1% Tween-20 (3 times, 5 min each time), the donkey anti-rabbit secondary antibody labeled with IRDye800 (1:5000; Rockland Immunochemicals, Boyertown, PA, USA) was added, followed by incubation at 4°C overnight and washing with TBST. A molecular Odyssey Infrared imaging system (Li-COR Biosciences, Lincoln, NE, USA) was used to scan the PVDF membrane and analyze the strips, using integrated optical density (IOD) as the relative protein content.

### Determination of HPSE mRNA expression by quantitative real-time PCR

Expression of HPSE mRNA was determined by real-time quantitative PCR on days 0, 1, 3, 7, 10, 14 and 21 of osteogenic differentiation. Total RNA was extracted from the cell sample using TRIzol according to the manufacturer’s instructions. The OD value and concentration were determined using a spectrophotometer. Total RNA (2 *μ*g) was transcribed to cDNA using an Omniscript RT kit. Primer 5 software was used to design the primers of the internal control glyceraldehyde 3-phosphate dehydrogenase (GAPDH) and the HPSE gene (Table I). The quantitative real-time PCR was conducted in the following conditions: 20 *μ*l reaction system (containing 1 *μ*l cDNA); 1 *μ*l upstream and downstream primers, respectively; 10 *μ*l EvaGreen^®^ qPCR Master Mix; and 7 *μ*l deionized water. The housekeeping gene GAPDH was used as the internal control.

### Statistical analysis

Statistical analysis was performed using SPSS 13.0 statistical software (SPSS, Inc., Chicago, IL, USA). Analysis of variance and t-test were performed to analyze the differences between the two types of rat. P<0.05 was considered to indicate a statistically significant difference.

## Results

### Expression of HPSE protein

**Figure 1:**
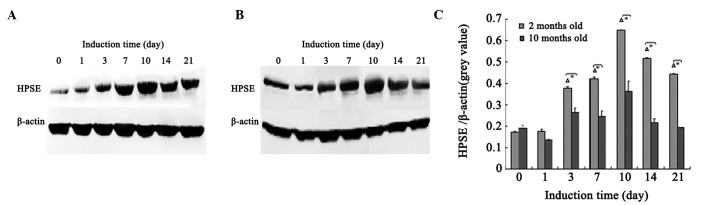
Heparanase (HPSE) protein expression in the osteogenic differentiation of rat marrow stromal cells (MSCs). (A) 2-Month-old rats; (B) 10-month-old rats; (C) histogram. ^Δ^P<0.05 compared with basal level; ^*^P<0.05 compared with 10-month-old rats.

The western blots in Fig. 1 show that, from day 3 of osteogenic differentiation of MSCs, the protein expression levels of HPSE in the 2-month-old rats were significantly increased compared with basal levels (days 0 and 1; P<0.05). HPSE protein expression reached a peak on day 10, followed by a gradual decline. On day 21, the HPSE protein expression level continued to be significantly higher than basal levels (days 0 and 1). A similar pattern was presented in the 10-month-old rats; however, the differences from basal levels were not significant (P>0.05). At each time point, the protein levels of HPSE in the 2-month-old rats were significantly higher compared with those in the 10-month-old rats (P<0.05).

### Expression of HPSE mRNA

**Figure 2:**
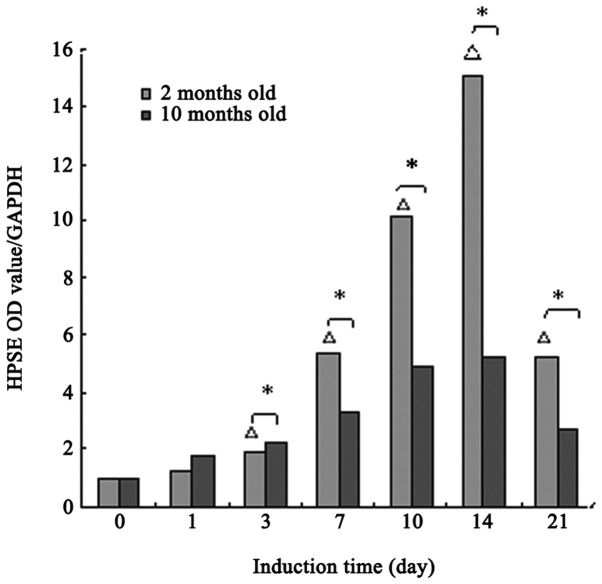
Heparanase (HPSE) mRNA expression in the osteogenic differentiation of rat marrow stromal cells (MSCs). ^Δ^P<0.05 compared with basal level; ^*^P<0.05 compared with 10-month-old rats. OD, optical density; GAPDH, glyceraldehyde 3-phosphate dehydrogenase.

As shown in Fig. 2, the mRNA expression levels of HPSE in 2-month-old rats began to increase on the third day, and was significantly different from basal levels (days 0 and 1; P<0.05). The expression levels remained significantly higher on day 21 (P<0.05). The same pattern was observed in 10-month-old rats; however, there were no significant differences from basal levels (P>0.05). As compared with HPSE protein level, the mRNA level reached a peak on day 14. This suggests that HPSE protein synthesis may be inhibited in the post-transcriptional modification stage.

## Discussion

Since Ogren and Lindahl first reported HPSE in mouse mast cells and demonstrated its digestive function on macromolecular heparin at specific sites (13), HPSE has been reported to be widely expressed in cells of normal tissues and malignant tumors. The gene sequences of the human HPSE gene were first determined in 1999, and the molecular structure, synthesis and action mechanism of HPSE were further studied (14,15). HPSE is the only known human endoglycosidase that plays an irreplaceable role in physiological and pathological processes. It has been a subject of intense research in molecular biology (16).

HPSE digests HS at specific sites, adjusts the release of HS-binding growth factors, including BMPs, FGFs and VEGF, regulates cell differentiation, adhesion and proliferation, and extracellular matrix reconstruction. In addition, HPSE has independent activity unrelated to enzyme function and directly activates corresponding receptors, increases AKT phosphorylation and participates in malignant tumor metastasis (17). HPSE is involved in pathological processes as follows: i) angiogenesis, metastasis and diffusion of myeloma and malignancy of the gastrointestinal tract and mammary gland (18); ii) tissue repair processes, including liver tissue regeneration, skin wound healing and hair regeneration (19,20); and iii) molecular biological mechanisms of kidney diseases, including diabetic nephropathy (21).

In addition, HPSE is involved in fracture healing and normal bone tissue formation. Saijo *et al*(11) studied a mouse model of fracture and detected HPSE mRNA in osteoclasts and precursor cells near the fracture site on day 5 after fracture. In the callus formation stage, a large amount of HPSE is synthesized in osteoclasts in cartilage callus absorption and neovascularization areas; it continues until the woven bone callus is transformed into a cortical bone callus. This indicates that HPSE is synthesized in osteoclasts in normal bone tissue and fracture sites. In the osteochondral border area, the synthesized HPSE activates cartilage absorption and bone formation and promotes the ossification of cartilage. It is considered that HPSE may be one of the key regulatory factors in bone tissue formation and regeneration. Smith *et al*(12) identified that the expression levels of HPSE mRNA in osteoporotic patients are significantly reduced compared with those in healthy volunteers. This indicates that HPSE mRNA expression is related to ALP activity. Additionally, when human osteoblasts are exposed to exogenous HPSE protein, the levels of phosphorylated histone H3 in osteoblasts are increased, suggesting that HPSE may adjust bone regeneration by regulating histone H3 phosphorylation. Kram *et al*(7) successfully cultivated HPSE-transgenic mice and identified that, compared with wild-type mice, the trabecular bone volume, cortical bone thickness and bone formation speed in HPSE-transgenic mice are significantly increased, respectively. This indicates that HPSE is involved in bone formation by regulating osteoblast activity.

In the present study, the expression of HPSE in the osteogenic differentiation of MSCs was investigated. Results show that from the third day of osteogenic differentiation, all HPSE protein and mRNA expression levels in 2-month-old rats were significantly increased compared with basal levels (days 0 and 1). The HPSE protein levels peaked on day 10 while HPSE mRNA levels peaked on day 14. This indicates that HPSE may be involved in the osteoblastic differentiation of MSCs. There were no significant differences of basal HPSE protein and mRNA expression levels between the 2- and 10-month-old rats; however, the responses of HPSE to osteogenic induction in the two ages of rat are different. The patterns of expression for the 10-month-old rats were similar to those of the 2-month-old rats; however, the differences compared with basal levels were not statistically significant. This indicates that the responses of HPSE to osteogenic induction in aged rats are reduced. HPSE may play an important role in bone tissue formation, which is consistent with results of the study by Smith *et al*(12).

HPSE is involved in the osteogenic differentiation of rat MSCs. The responses to osteogenic induction in aged rats are weaker compared with those in young rats, which may be related to the decline in osteogenic differentiation ability. The specific mechanism of participation of HPSE in osteogenic differentiation is worthy of further investigation. This is likely to contribute to an in-depth understanding of fracture healing and osteoporosis pathogenesis, as well as create conditions for exploring more effective clinical treatment methods.

## Figures and Tables

**Table I:** Primers and amplification fragment length.

Gene	Upstream primer (5′-3′)	Downstream primer (5′-3′)	Amplified fragment length (bp)
GAPDH	GGCATCCTGGGCTACACT	CCACCACCCTGTTGCTGT	163
HPSE	CGGTTCTGACGGACTGCTT	AAAACCCATAGGAAAAGGCG	146

GAPDH, glyceraldehyde 3-phosphate dehydrogenase; HPSE, heparanase.
